# A Guide to the Clinical Examination and Treatment of Sick Children

**Published:** 1899-01

**Authors:** 


A GUIDE TO THE CLINICAL EXAMINATION AND TREATMENT OF SICK CHILDREN. By JOHN THOMSON, M. D., Extra Physician to the Royal Hospital for Sick Children, London; Lecturer on Diseases of Children, Edinburgh School of Medicine. In one crown 8vo volume of 350 pages, with fifty-two illustrations. Cloth, $1.75 net. Philadelphia and New York: Lea Brothers & Co., Publishers.

To a young medical man or woman, inexperienced in examining children, lacking through ignorance as well as inexperience the tact requisite to make an examination successfully, and above all scarcely knowing how a well baby looks and acts, let alone a sick one, we cannot commend too warmly Dr. Thomson's book. The older physican who has had a considerable practice among children has already learned for himself some of the lessons it teaches, but, nevertheless, it is a book which he will enjoy reading, and one whose perusal will repay him. The author devotes a large part of the book to the examination of the child. Then follows chapters on nursery hygiene, infant feeding, therapeutics, including various agents other than drugs, as baths, packs, poultices, and the like, the indications for their use and the manner of employing them; and on food disorders, including rickets and scurvy. Barring the chapter on infant feeding, which hardly satisfies our American notions on the subject, there is no unfavorable criticism to be made of the text. In its particular mission, that of familiarising the practitioner with the normal as well as the abnormal conditions of infancy and childhood, we know of nothing which approaches it in value.

